# PTSD and opioid use: implications for intervention and policy

**DOI:** 10.1186/s13011-020-00264-8

**Published:** 2020-03-16

**Authors:** Lucia Dahlby, Thomas Kerr

**Affiliations:** 1 British Columbia Centre for Substance Use, 400-1045 Howe Street, Vancouver, BC V6Z 2A9 Canada; 2 Department of Medicine, University of British Columbia, St. Paul’s Hospital, 608-1081 Burrard Street, Vancouver, BC V6Z 1Y6 Canada

**Keywords:** Posttraumatic stress disorder, Opioid use disorder, Overdose epidemic, Mental health, Public policy

## Abstract

**Background:**

North America remains in the midst of an escalating opioid overdose epidemic, largely driven by the influx of synthetic opioids such a fentanyl and related analogues. High rates of mental illness among substance-using populations have been well documented; in particular, opioid-using individuals suffer from high rates of PTSD. Despite the devastating disease burden of both PTSD and OUD, especially within the context of the current opioid overdose epidemic, treatment options and outcomes remain suboptimal.

**Main body:**

Comorbid PTSD-OUD is often complex and inextricably intertwined, thereby impeding effective diagnosis, assessment and early intervention. Best outcomes occur when treatment addresses both comorbidities simultaneously, known as parallel or integrative approaches. Despite these findings, affected individuals often do not receive adequate or equitable access to healthcare. The WHO recommends that public spending for both mental and physical aspects of healthcare be equitable to the burden of disease. Despite these recommendations mental healthcare services remain chronically underfunded in Canada. The Mental Health Parity Act is a call for the Canadian government to implement equitable public spending on all aspects of healthcare. Furthermore, prohibitory legislative practices serve to marginalize substance-using populations thereby increasing the likelihood of exposure to traumatic violence and other associated harms.

**Conclusion:**

Efforts are now needed to address regulatory drug-use frameworks and public healthcare policies that perpetuate these inequalities. Alternative regulatory frameworks for drugs and mental health parity should be implemented and evaluated in an effort to reduce violence, trauma and ultimately opioid-related overdose deaths.

## Background

North America remains in the midst of an escalating opioid-related overdose epidemic. From 2016 to 2017 Canada saw a 33% increase in opioid-related overdose deaths, and in 2018 alone these fatalities represented 11.8 per 100,000 of the Canadian population [1]. This epidemic also continues to place undue burden on the healthcare system, with opioid-related overdose emergency room visits increasing by 27% since 2013 [1].

Although there is evidence to suggest a role of overprescribing and misuse in driving the opioid overdose epidemic, it is becoming increasingly clear that illicit synthetic opioids, such as fentanyl and related analogues, are accounting for a growing proportion of deaths throughout North America. In Canada, fentanyl was found to be involved in 73% of apparent opioid-related overdose deaths in 2018 [1]. These data illustrate the magnitude of synthetic opioid contamination into mainstream drug markets and have positioned fentanyl as a major public health concern.

Posttraumatic stress disorder (PTSD) and mental illness have been found to commonly co-occur, in particular, a growing body of literature points to high rates of PTSD among opioid-using populations [2–4]. Despite high prevalence rates of PTSD among SUD/OUD populations, patients seeking treatment for substance use are rarely assessed for trauma or offered PTSD-based interventions alongside SUD treatments [2]. Similarly, OAT clinics, a frequent point of contact among OUD individuals and healthcare providers, seldom provide regular trauma screenings, PTSD assessments or PTSD-based interventions alongside OUD treatment [2].

From a public health perspective, these deficits indicate a need for greater coordination of care among service providers, as well as, increased integration across various aspects of healthcare. From a socio-structural perspective, high rates of traumatization among SUD populations and the fentanyl/opioid overdose epidemic suggest a need to implement and evaluate novel regulatory substance-use frameworks in order to address the escalation in overdose deaths.

## Main text

### PTSD-0UD comorbidity

Extant literature consistently indicates a high incidence of PTSD among substance using populations, with lifetime prevalence rates among SUD individuals ranging from 26 to 52% [2]. Relatively less research has focused on rates of PTSD among OUD individuals. However, preliminary evidence suggests that rates are equally high; among OUD populations, 41% have a lifetime history of PTSD and 33.2% meet criteria for a current PTSD diagnosis, representing the highest rate of PTSD among substance users [4].

To account for this comorbidity a ‘self-medication hypothesis’ has been proposed, in which opioid use alleviates PTSD symptom severity, thereby placing individuals at an increased risk of habitual self-administration and ultimately addiction [2, 3]. In support of this hypothesis, findings indicate that PTSD patients have a significantly higher subjective pain rating and rate of prescribed opioid use than non-PTSD patients. Overstimulation of the noradrenergic system in individuals with PTSD and the sedating effects of opioids may underlie this mechanism, as a reduction of PSTD symptom severity has been reported following acute morphine administration [2, 3]. Additionally, opioid withdrawal further activates the noradrenergic system, therefore, leading to habitual self-medication by means of negative reinforcement [3].

Alternatively, there is also evidence to suggest the inverse, in which substance use disorder (SUD), specifically associated social-structural exposures, predispose individuals to higher rates of traumatization thus precipitating the development of PTSD [2, 5]. Under current regulatory frameworks, in which substance-use is criminalized, disputes within drug markets are often resolved through violence, and to cover the cost of drugs substance users often rely on income generating activities such as sex work and drug dealing, that carry a high risk of violence and trauma [2]. Accordingly, substance-using populations are 5.91 times more likely to experience traumatization than non-SUD populations [5], therefore placing individuals who already suffer from one condition (SUD) at an increased risk of developing the other (PTSD). Furthermore, being diagnosed with both conditions appears to increase the risk and severity of the other: substance use increases the risk of re-traumatization, and re-traumatization increases the risk and severity of subsequent substance use, therefore perpetuating the PTSD-substance use comorbidity cycle [4].

### PTSD-OUD treatments

The scale-up of various pharmacological treatments for substance use disorders, such as opioid agonist treatment (OAT), has been a critical response to the overdose epidemic throughout North America. Increased OAT access is associated with higher treatment retention and decreased illicit heroin use in comparison to non-pharmacological treatments, as well as a decrease in opioid-related overdose deaths for both buprenorphine and methadone [6]. Research regarding the influence of PTSD comorbidity on OAT outcomes has yielded inconclusive, and often contradictory, results. Recent studies have found compelling evidence that PTSD symptom severity may undermine OAT retention and outcomes; such that with every 10% increase in PTSD symptom severity there is an associated 36% increase risk in OAT interruption, and re-traumatization is associated with double the risk of OAT interruption. Treatment interruption is generally operationalized as absence from methadone administration for at least 1 week in a given month, and often used as an indicator of treatment adherence. Furthermore, patients who dropped out of OAT endorsed greater PTSD symptom severity than those who were able to adhere to the treatment regime [4]. Conversely, other studies have failed to find a significant association between PTSD and OAT adherence, retention or outcomes [4]. Moreover, recent research suggests that OAT can be equally effective in reducing illicit opioid use, regardless of PTSD comorbidity [3, 4]. Despite these divergent results, it is generally agreed upon that while OAT can be an effective treatment for OUD, it appears to offer little therapeutic effect on PTSD symptom severity. Additionally, treatment that targets SUD only is associated with worse recruitment, retention, adherence and outcomes, as well as, shorter periods of abstinence post-treatment among comorbid PTSD-SUD individuals [2].

Overall, evidence suggests that the best outcomes occur when treatment approaches include both trauma-focused psychological interventions alongside or combined with treatments for SUD/OUD, otherwise known as parallel and integrative treatment approaches, respectively [2–4]. Parallel treatment involves the individual delivery of a trauma-focused therapy alongside, but separate from, an evidence-based SUD treatment; this approach is considered most effective [2]. Alternatively, integrated treatment involves both trauma-focused and substance-use-focused therapies being delivered simultaneously, or combined into a single treatment; this approach has received comparatively less evidence-based support [2].

However, as mentioned earlier, SUD treatments, especially OAT clinics, often do not assess or screen for PTSD, and rarely provide PTSD-based interventions. As such, patients seeking treatment for these co-morbid conditions tend to get passed between various services or referred to other providers with little coordination of care [2]. Furthermore, the complex nature of comorbid patients makes assessment, diagnosis and treatment increasingly difficult for clinicians. Patients with both disorders tend to present more severe clinical profiles and are at increased risk for other psychiatric difficulties. In regard to OUD, assessment and diagnosis is further complicated by symptoms of opioid withdrawal, which can mimic the presentation of PTSD symptomology (i.e. hyper-vigilance and increased startle response) [2, 3].

Efforts are now needed to address trauma, PTSD and mental health in general among people who use drugs. Evidence-based trauma-focused screenings, assessments and interventions should become widely implemented and coupled with the delivery of OAT. Despite the necessity for increased mental health and trauma screenings among substance using populations, many do not receive adequate health care; with 40% of substance using individuals suffering from a comorbid mental health disorder, only 6.8% of these individuals receive treatment for both conditions and 52% receive no treatment at all [7]. The various health and socio-demographic disparities among this population reflect systemic barriers at both the micro and macro levels of public healthcare. Important questions are now raised about what can and should be done to break down these barriers to care and most effectively address this complex comorbidity.

### Existing barriers and future recommendations

A major barrier facing Canadians who suffer from complex co-occurring mental illness, such as PTSD-OUD, is the continued and chronic underfunding of community-based mental health services, as well as, the over-reliance on acute, intensive and costly forms of care, such as psychiatrists and hospitals. The World Health Organization (WHO) recommends that public spending be in proportion to the burden of illness for both mental and physical forms of healthcare [8]. Despite these recommendations the ratio of disease burden to public spending for mental health services in Canada remains disproportionate, at 3:1, respectively [9]. Furthermore, Canada devotes only 7.2% of its healthcare budget on mental health services, the lowest allocation of funds among all G7 countries [8]. The apparent disparities between mental and physical forms healthcare suggest that mental health continues to be chronically underfunded, consistently devalued, and remains highly stigmatized within Canada.

Recently, Canada’s healthcare system has come under scrutiny with a call for the introduction of a Mental Health Parity Act. Parity refers to the notion that mental health should have equal status to physical health, in terms of funding, access and quality of healthcare services. The proper implementation of a Mental Health Parity Act would allow the allocation of more public funding for evidence-based therapies, improve quality of care through a continuum of integrated services, and address stigma and discrimination through equitable access to care [8]. Therefore, in order to specifically address PTSD-OUD comorbidity, and mental health, addiction and the opioid overdose epidemic, more generally, Canada should implement the Mental Health Parity Act.

Increased access and funding to evidence-based, integrated mental health services is a critical component in addressing health disparities at the individual level. However, these issues are the outcome of larger, systemic, regulatory frameworks. High rates of trauma, in particular violence, persist among substance-using populations due to the fact that illicit drugs remain criminalized. Therefore, novel regulatory approaches, such as the decriminalization of illicit drug use, should also be widely evaluated and implemented in an effort to reduce violence, trauma, PTSD and in turn, overdose rates. Canada should now look to other governing bodies as an example of such regulatory frameworks, where, in places like Portugal, drug decriminalization has resulted in the reduction of problematic drug use, drug-related harms, drug-related criminality and overcrowding within the criminal justice system [10].

## Conclusion

High PTSD-OUD comorbidity rates and poor treatment outcomes discussed in this commentary suggest that current public health policies, regarding mental health and substance-use, may be ineffective and ultimately inadequate. The underfunding of mental health services prevents affected individuals from receiving equitable access to care and contributes to the devaluation of mental illness within the healthcare system. The Mental Health Parity Act, which requires mental health services to be funded on par with physical healthcare services, may help to break down this barrier and bridge socio-demographic health disparities. Furthermore, current prohibitory drug-use frameworks marginalize substance-users; these individuals are repeatedly exposed to unregulated street drug markets, which carries a high-risk of violence. Consequently, high-rates of traumatic exposure, particularly due to violence, persist among substance-using populations. Governing bodies should now consider alternative approaches to drug-use regulation, such as decriminalization. Decriminalization, which has been shown to reduce violent crime, substance-use-related harms and overdose, would therefore address both high traumatization rates and the opioid overdose epidemic, simultaneously. Comorbid PTSD-OUD is an increasingly complex issue, and while there is currently no definitive evidence-based solution, it is clear that existing frameworks bear various limitations and shortcomings. Innovative public health policies must therefore be widely discussed, evaluated and implemented in order to bridge these health disparities and address the overdose epidemic in Canada.

## Data Availability

Not applicable.

## Abbreviations

OAT: Opioid agonist therapy
OUD: Opioid use disorder
PTSD: Posttraumatic stress disorder
SUD: Substance use disorder
WHO: World Heal Organization
